# Response of Blood Pressure to Renal Denervation Is Not Associated With Genetic Variants

**DOI:** 10.1161/HYPERTENSIONAHA.124.23393

**Published:** 2024-11-21

**Authors:** Christian Delles, Roland E. Schmieder, Rónán Daly, Dennis Kannenkeril, Agnes Bosch, Lucas Lauder, Michael Kunz, Michael Böhm, Graham Hamilton, Raphael S. Schmieder, Axel Schmid, Pawel Herzyk, Felix Mahfoud

**Affiliations:** School of Cardiovascular and Metabolic Health (C.D.), University Hospital Erlangen, Friedrich-Alexander University Erlangen-Nürnberg, Germany.; Glasgow Polyomics, Wolfson Wohl Cancer Research Centre (R.D., G.H., P.H.), University Hospital Erlangen, Friedrich-Alexander University Erlangen-Nürnberg, Germany.; University of Glasgow, UK. Department of Nephrology and Hypertension (R.E.S.), University Hospital Erlangen, Friedrich-Alexander University Erlangen-Nürnberg, Germany.; Institute of Radiology (R.S.S., A.S.), University Hospital Erlangen, Friedrich-Alexander University Erlangen-Nürnberg, Germany.; Department of Internal Medicine III, Cardiology, Angiology, Intensive Care Medicine, Saarland University Hospital, Homburg, Germany (L.L., M.K., M.B., F.M.).; Department of Cardiology, University Heart Center Basel, University Hospital Basel, Switzerland (L.L., M.K., F.M.).; Institute for Medical Engineering and Science, Massachusetts Institute of Technology, Cambridge (F.M.).

**Keywords:** blood pressure, catheters, genetic profile, genomics, hypertension

## Abstract

**BACKGROUND:**

Renal denervation lowers blood pressure (BP) in patients with uncontrolled hypertension. We conducted an unbiased genomic screen to identify genetic variants that may associate with BP response to renal denervation (RDN).

**METHODS:**

Patients (n=268) with uncontrolled resistant hypertension (baseline BP, 166±21/90±15 mm Hg) who underwent endovascular RDN using the Symplicity catheter (Medtronic, Inc, Santa Rosa, CA) were included. Reduction in 24-hour ambulatory systolic BP was assessed at 6 months and divided into 2 groups: above and below the median response of 6.0 mm Hg, taking preintervention 24-hour ambulatory BP and regression to the mean into account. Whole exome sequencing assessing 249 669 variants, was conducted using Illumina NovaSeq technology read on a NovaSeq S4 Flow Cell device.

**RESULTS:**

We did not identify individual gene variants associated with BP response following RDN. These findings were confirmed after adjustment for sex and in a sensitivity analysis looking at tertiles of BP response. We also explored specific variants in *AGT*, *ADD1*, ADRB1, *ADRB2*, and *SCNN1A* that have been proposed as potential candidate genes for response and found no association (all *P*>0.13). Gene ontology analysis of variants across the 2 responder groups highlighted differences in biologic processes such as cell adhesion and molecular function such as protein tyrosine kinase activity.

**CONCLUSIONS:**

The response to RDN, in terms of 24-hour BP reduction, was not associated with the genetic profile of patients with resistant hypertension. These data do not support the use of a genetic score to identify potential responders to RDN.

**NOVELTY AND RELEVANCE**

**What Is New?**

This study has not found an association between genetic variants and blood pressure response to renal denervation.

**What Is Relevant?**

Catheter-based renal denervation is a therapeutic option for patients with treatment-resistant hypertension. However, it is currently not possible to predict an individual’s blood pressure response to renal denervation.

**Clinical/Pathophysiological Implications?**

This study does not support genetic testing to aid decisions about renal denervation in the management of patients with treatment-resistant hypertension.

Catheter-based renal denervation (RDN) has been introduced into clinical trials and clinical practice more than a decade ago. Long-term safety and long-term efficacy have been demonstrated and meta-analyzed recently.^1–3^ Several position papers and guidelines recommend RDN as an adjunct treatment approach in patients with uncontrolled resistant hypertension.^4–6^ In November 2023, the Symplicity Spyral radiofrequency and the Paradise ultrasound RDN system received premarket approval from the U.S. Food and Drug Administration as an adjunctive blood pressure (BP)-lowering treatment in patients with hypertension in whom BP remains above treatment targets despite lifestyle modifications and antihypertensive pharmacotherapy.

The average BP-lowering effects of RDN, compared with sham, was 4.4 mm Hg and 6.6 mm Hg for 24-hour and office systolic BP (SBP), respectively. However, there is a large interindividual variability in BP change. Several potential predictors of response to RDN have been suggested including renal artery anatomy^7^; baseline BP traits such as BP variability^8^; clinical and demographic conditions such as gender, presence of diabetes or obesity^9,10^; pathophysiological factors such as skin sodium levels,^11^ activation of the renin-angiotensin-aldosterone system,^12^ and the sympathetic nervous system (SNS)^13^; and presence and severity of organ damage such as vascular stiffness^14–16^ and, particularly, invasive pulse wave velocity and aortic distensibility.^17^ While statistically significant associations were sometimes found, replication in independent datasets has not been performed or revealed inconsistent findings, with the exception of baseline BP, which is a strong predictor of response for all antihypertensive regimens.^18^

The effect of RDN is in the first instance, due to the modulation of sympathetic signaling in the kidney^19^ but it directly and indirectly extends to other mechanisms that play a key role in BP regulation. For example, unilateral RDN in a preclinical model of renovascular hypertension has the potential to also interfere with reactive oxygen species production and renin-angiotensin-aldosterone system activity.^20,21^ It is therefore reasonable to assume that the BP response to RDN depends also on mechanisms other than SNS activity and that predictors of response could include a wide range of BP-related pathways.

Hypertension is a condition of multifactorial and polygenic origin. Over 1000 genetic loci have been found to be robustly associated with hypertension,^22^ and our understanding of the underlying mechanisms paves the way to a precision medicine-led approach to BP management.^23^ Genetic variability in the activity of the SNS is well established,^24,25^ and RDN reverses a genetic salt-sensitive form of hypertension in a rodent model.^26^ A role of the genetic makeup in the response to RDN has therefore been proposed.^25^

Herein, we applied a nonbiased genomic approach to study the association between genetic factors and response to catheter-based RDN by investigating the association between genetic variants determined by exome sequencing and BP changes following RDN.

## Methods

### Data Availability

The data that support the findings of this study are available from the corresponding author upon reasonable request.

### Patients and RDN Procedure

In this investigator-initiated, prospective study, adults with uncontrolled hypertension who underwent catheter-based endovascular RDN in 2 academic centers (Erlangen-Nürnberg, Germany; and Homburg, Germany) were included. All patients had resistant hypertension, which was defined as office BP >140/90 mm Hg and 24-hour ambulatory BP >130/80 mm Hg despite treatment with at least 3 antihypertensive drugs. All patients had to be on a stable antihypertensive drug regimen, without any changes in drug doses or regimen adjustments, for at least 2 months. No medication changes were allowed during follow-up. Exclusion criteria were secondary causes of arterial hypertension (except chronic obstructive sleep apnea), pregnancy, type 1 diabetes, and significant renal artery abnormalities (main renal arteries <4 mm in diameter or <20 mm in length, hemodynamically or anatomically significant renal artery abnormality or stenosis in either renal artery, history of renal artery intervention including balloon angioplasty, or stenting, multiple main renal arteries in either kidney).

Patients who entered the present genetic study were drawn from randomized controlled trials and registries from the 2 sites. In Erlangen, patients with resistant hypertension were treated between 2010 and 2018 within the single-center, investigator-initiated Renal Denervation in Treatment Resistant Hypertension trial (REGISTRATION: URL: https://www.clinicaltrials.gov; Unique identifier: NCT01687725). Patients were reinvited between 2019 and 2020 to provide samples for genetic analyses as stated in REGISTRATION: URL: https://www.clinicaltrials.gov; Unique identifier: NCT04321044. In Homburg, patients were recruited from 2010 to 2018 into the Homburger Hypertonie Register and as part of REGISTRATION: URL: https://www.clinicaltrials.gov; Unique identifier: NCT01888315. DNA from Erlangen and Homburg patients was extracted in parallel at the Erlangen site.

RDN was performed by experienced interventionalists using the Symplicity catheter (Medtronic, Inc).^13,27^ In brief, all procedures were performed via femoral access with standard endovascular techniques, and the renal arteries of both sides were treated in 1 session. The radiofrequency catheters were advanced into each renal artery, guided by angiography. At least 4 radiofrequency ablations (energy delivery for 120 seconds each), controlled and regulated by a radiofrequency generator, were longitudinally and rotationally applied within the lengths of each renal artery. Diffuse visceral pain during the procedure was managed with analgesics and narcotics.

The study adhered to the principles of the Declaration of Helsinki and Good Clinical Practice guidelines. The study protocol was approved by the local ethics committees of the 2 participating centers (CRC2019GBA and 142/10). All participants provided written informed consent. The study was registered at www.clinicaltrials.gov (REGISTRATION: URL: https://www.clinicaltrials.gov; Unique identifier: NCT04321044).

### 24-Hour Ambulatory BP Measurement

All participants underwent 24-hour ambulatory BP monitoring before RDN and 6 months after the procedure. Ambulatory 24-hour BP measurements were taken with an automatic portable device that has been validated (eg, Spacelab no. 90207; Redmont, CA).^18^ BP readings were taken every 15 minutes throughout the day and every 30 minutes throughout the night. Cuff size was adjusted to the circumference of the upper arm in each patient. We originally aimed for a convenience sample of 300 patients, but having excluded those with incomplete 24-hour ambulatory BP data, we arrived at a total of 264 included patients.

### Definition of Response

Response of 24-hour ambulatory BP was assessed after 6 months and divided into 2 groups, above and below the median response of 6.0 mm Hg, taking preintervention 24-hour ambulatory BP and regression to the mean into account.^18^ In other words, the change in 24-hour ambulatory SBP was plotted against baseline 24-hour ambulatory SBP, and a regression line was drawn. Patients whose BP changes were above the regression line were classified as nonresponders whereas those whose BP changes were below the regression line were classified as responders (Figure S1). By putting response into relation to baseline BP, this approach takes regression to the mean and greater expected effects in patients with greater baseline BP into account^28^ (Table S1).

### Exome Sequencing

Genomic DNA was extracted from 2 to 5 mL of whole blood by standard methods using a commercially available kit (QIAamp Blood Midi Kit; QIAGEN GmbH, Hilden, Germany). Whole exome sequencing assessing 249 669 variants was conducted using Illumina NovaSeq technology read on a NovaSeq S4 Flow Cell device (Illumina, Inc, San Diego, CA). Reads were analyzed by a standardized variant-calling pipeline based on the Genome Analysis Toolkit (Figure S2), as previously described in detail elsewhere.^29^ Read quality was overall excellent, with the 8 poorest-performing samples still covering 30 reads per base for 80% of the exome (Figure S3).

### Data Analysis and Statistics

Variant annotation was conducted using the Ensembl Variant Effect Predictor.^30^ Genomic data were analyzed using principal component analysis followed by individual logistic regressions to model the association of variants with phenotype where appropriate. Principal component analysis was performed using the snpgdsPCA function of the R SNPRelate package, taking the first 5 eigenvectors.^31,32^

Genome-wide association was studied by examining the distribution of *P* values as proposed by Storey and Tibshirani.^33^

Gene ontology analysis was conducted using the gprofiler2 R package, particularly the gost function, for gene list functional enrichment. All genes with an uncorrected *P*<0.05 were input to test for functional enrichment.

Clinical parameters were analyzed by Student *t* test for continuously distributed data and χ^2^ test for categorical data using SPSS software (version 27; IBM, Armonk, NY). Two-tailed *P*<0.05 was considered significant.

To examine the sensitivity of results to sample size, a post hoc power analysis was conducted by treating the results as a pilot study and resampling the data with replacement, with resample sizes between 100 and 25 600. This procedure was conducted 30× per resample size, with the logistic regression analysis being conducted as mentioned above, the proportion of significant results being recorded, and these results averaged over each resample size. Results are shown in Figure S4. The analysis indicates that given the effects from the measured data, a total sample size of 4219 would be needed for a power of 0.8 on 80% of the features.

## Results

### Patients

A total of 268 patients were included in the study. The Table depicts the clinical data of responders (mean SBP change, −23±16 mm Hg) and nonresponders (mean SBP change, +1±11 mm Hg). No statistically significant differences in clinical and demographic parameters between the 2 groups were found, with the exception of glycated hemoglobin (HbA_1__c_), which was greater in nonresponders compared with responders. When the cohort was divided into tertiles of BP change focusing on extreme response, there were also no differences between responders and nonresponders except for HbA_1c_ (data not shown).

**Table.**
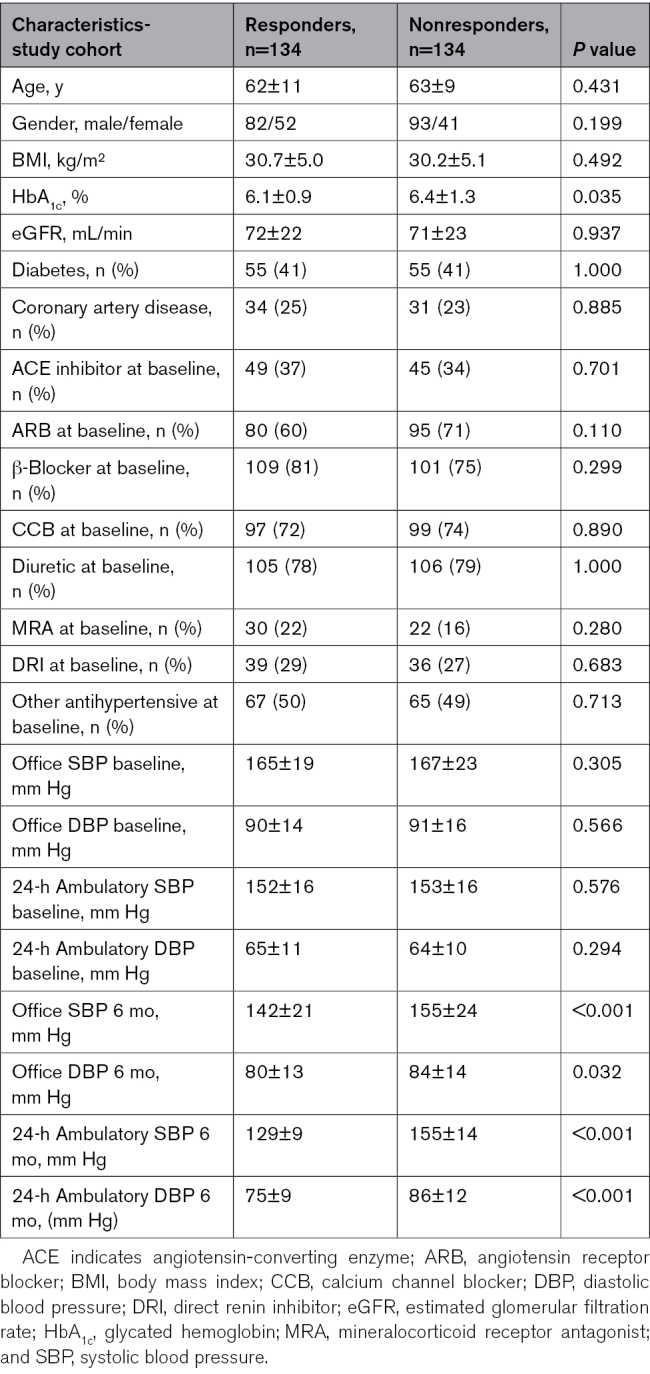
Characteristics of the Study Cohort

### Genetic Variants

Exome sequencing revealed a total of 249 669 genetic variants, the majority of which were single nucleotide variants, and most were missense variants (Figure S5).

### Principal Component Analysis

**Figure 1.**
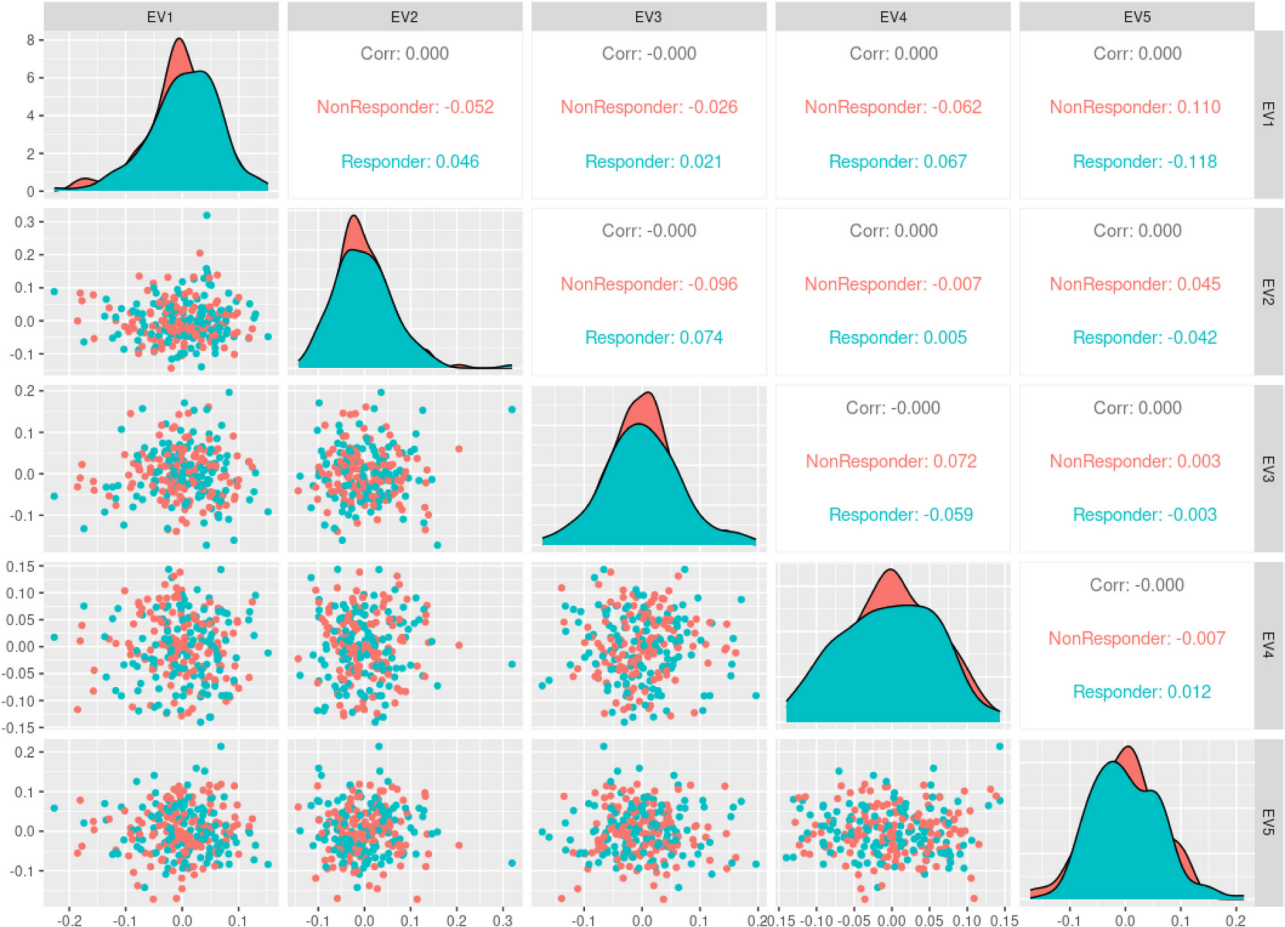
**Principal component analysis scatter plot matrix.** The projection of the data for the first 5 eigenvectors is plotted against each other. The plot shows no separation between the responders and nonresponders across these components. Axes are unitless. Corr indicates correlation; and EV, eigenvector.

We conducted a principal component analysis to unravel any separation of responders and nonresponders based on any of the genetic variants. There was no statistically significant separation between the 2 groups for any of the first 5 eigenvectors (Figure 1).

### Genome-Wide Association

**Figure 2.**
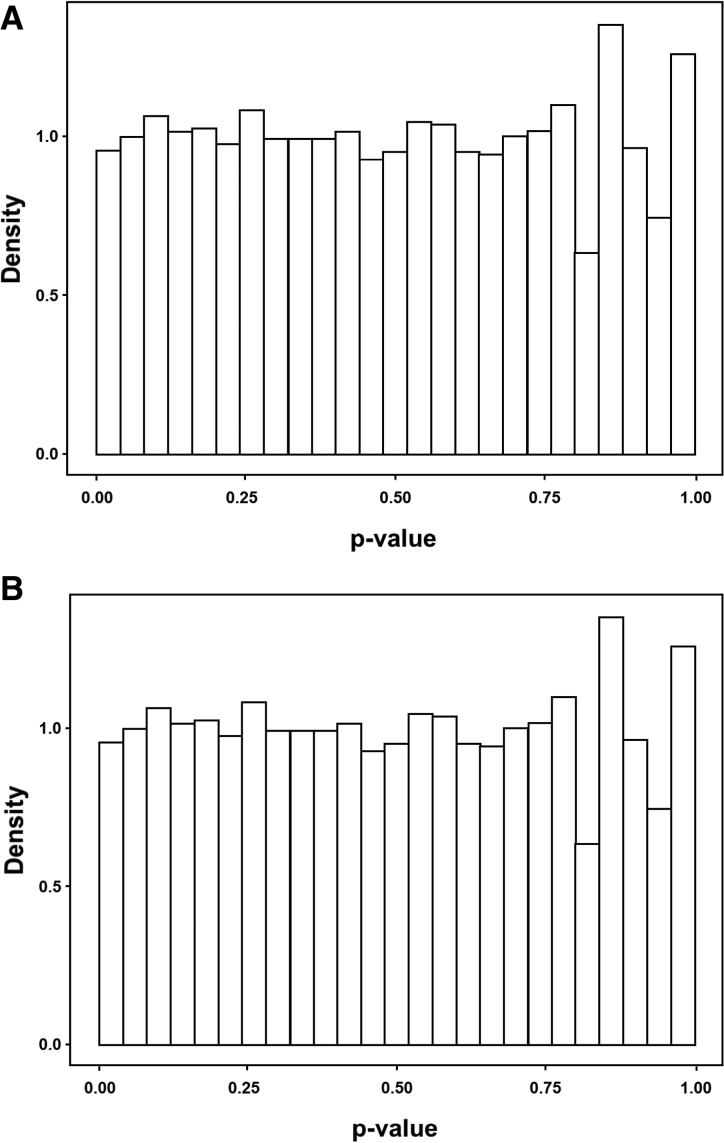
**Distribution of *P* values.** Genome-wide association was studied by examining the distribution of *P* values as proposed by Storey and Tibshirani.^32^ **A**, Distribution of *P* values in unadjusted data. **B**, Distribution of *P* values in data adjusted for sex. Overall, no evidence was found to reject the null hypothesis of no genome-wide association with blood pressure response to renal denervation.

We plotted a density histogram of *P* values associated with genetic variants. These were roughly uniformly distributed and did not deviate from the estimate of the proportion of null *P* values, indicating no significant genome-wide association with response to RDN (Figure 2).

### Gene Ontology Analysis

**Figure 3.**
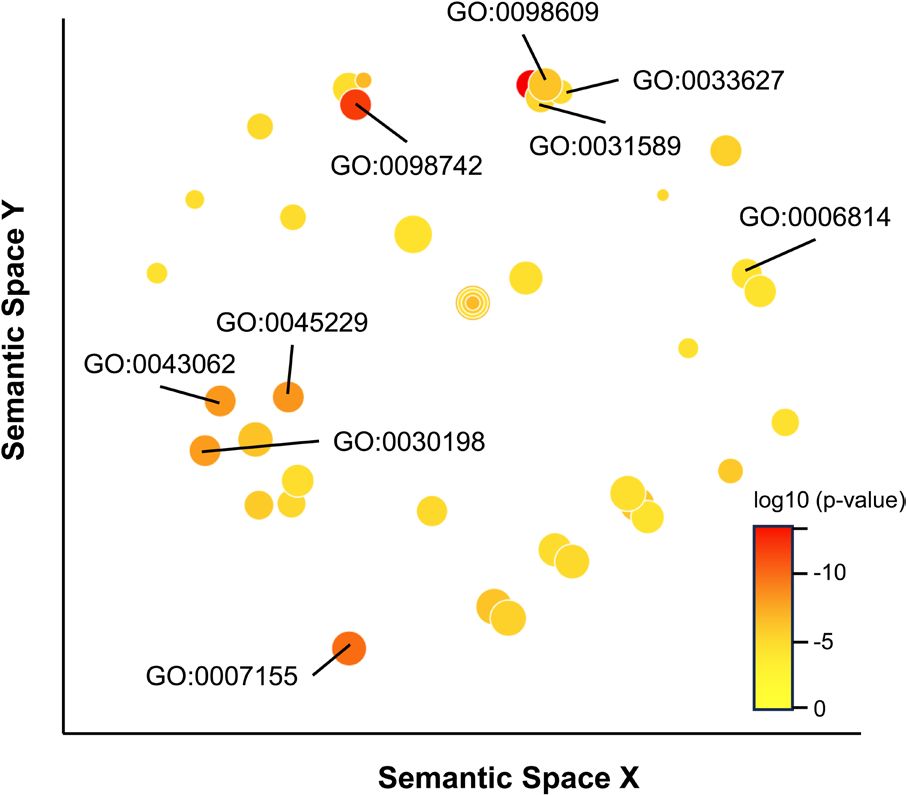
**Gene ontology analysis of differentially regulated biologic processes.** The figure illustrates gene ontologies (GOs) and their relationships within semantic spaces. Three key processes are labeled: cell adhesion (GO:0007155, cell adhesion; GO:0031589, cell-substrate adhesion; GO:0098609, cell-cell adhesion; GO:0098742, cell-cell adhesion via plasma-membrane adhesion molecules; GO:0033627, cell adhesion mediated by integrin), sodium transport (GO:0006814, sodium ion transport), and extracellular matrix (GO:0030198, extracellular matrix organization; GO:0043062, extracellular structure organization; GO:0045229, extracellular encapsulating structure organization). Detailed data are provided in Table S2. *P* values are illustrated by bubble color. Bubble size represents LogSize (ie, log10 number of annotations for GO term identifier). Analysis was conducted using the GO Enrichment Analysis tool (https://geneontology.org/) with standard settings. Enrichment analysis and graphical output were conducted with REVIGO (http://revigo.irb.hr/) using H. sapiens reference data. The *x* and *y* axis units were removed as they are arbitrary and nonlinear in REVIGO.

Gene ontology analysis of biologic processes revealed differences in the regulation of processes such as cell adhesion and sodium ion transport between responders and nonresponders (Figure 3; Table S2). Further analyses of differentially regulated cellular components (Table S3) and molecular functions (Table S4) revealed further features linked to the genetic signals and potentially involved in the response to RDN, including cell-cell junction components, brush border components, constituents of the extracellular matrix, and calcium-ion binding.

### Single Nucleotide Polymorphisms

While our study was designed to genotype a large number of variants and examine genome-wide significance it naturally covered variants in potential candidate genes, such as *AGT* (rs699, rs5051), *ADD1* (rs5961), *ADRB1* (rs1801252, rs1801253), *ADRB2* (rs1042713, rs1042714), and *SCNN1A* (rs2228576). None of these single nucleotide polymorphisms achieved even nominal significance in the comparison between responders and nonresponders (all *P*>0.10).

### Sensitivity Analysis

We used a more stringent definition of response versus nonresponse to RDN by comparing the top with the bottom tertile of response. In this analysis, principal component analysis also did not reveal genetic differences between responders and nonresponders, and no genome-wide association was found (Figures S6 and S7).

## Discussion

In this study, we used exome sequencing to interrogate the association between genetic variants and BP response to RDN in patients with uncontrolled resistant hypertension. There was no genetic signal with a significant association with future BP response, neither on a genome-wide nor on a single-variant basis. Gene ontology analysis showed, however, that cellular processes, cellular components, and molecular functions such as cell adhesion and sodium ion transport were different between responders and nonresponders.

According to the 2023 European Society of Hypertension guidelines, RDN can be considered as a treatment option in patients with an estimated glomerular filtration rate >40 mL/min per 1.73 m^2^ who have uncontrolled BP despite the use of antihypertensive drug combination therapy, or if drug treatment elicits serious side effects and poor quality of life, or as an additional treatment option in patients with true resistant hypertension and preserved renal function.^4^ Meta-analyses of sham-controlled trials have shown that, compared with sham, RDN reduced 24-hour and office SBP by 4.4 mm Hg and 6.6 mm Hg, respectively. The 24-hour and office diastolic BP paralleled these findings, and the safety profile was excellent.^34^ These changes are clinically meaningful, as a reduction in office SBP by 5 mm Hg has been shown to translate into a relative reduction in stroke by 13%, heart failure by 14%, and major cardiovascular complications by 10%.^35^

The invasive nature of the procedure and the relatively high initial costs have led to numerous attempts to identify potential predictors of BP response to RDN. This would not only allow precise prediction of the risk-benefit-ratio and inform patient selection but also facilitate the shared decision process, which is important for interventions in general and for invasive procedures in particular.^4^ One would assume that the efficacy of a procedure aimed at modulating the activity of the SNS would be driven by factors that determine SNS activity, such as sex, age, and obesity, but also factors that associate with SNS activity, such as heart rate and BP variability, or factors that measure SNS activity directly such as noradrenaline spillover and microneurography techniques. One may also speculate that patients with advanced hypertension-mediated organ damage, and in particular with advanced vascular stiffening, as well as patients with comorbidities such as diabetes, will respond differently and presumably less to RDN compared with those with shorter durations of hypertension, absence of overt organ damage, and absence of comorbidities. In fact, many of such factors have been found to correlate with BP response to RDN, at least in small case series, but their predictive value as a clinical tool is modest and has often not been replicated in independent and larger datasets.^18^

It should also be mentioned that the precise BP-lowering mechanism of RDN remains incompletely understood and includes effects beyond the renal SNS.^36,37^ We have therefore selected an unbiased approach by studying the predictive value of genetic variants determined by exome sequencing for BP response to RDN. While this approach is different from a hypothesis-driven approach into specific genetic variants and has lower power on an individual variant level, it would have shown any meaningful genome-wide signal that would be worth examining in further validation and mechanistic studies. We presented multiple lines of evidence of the absence of such association at the genome level. In this context, our gene ontology data are, however, of interest as they demonstrate that responders and nonresponders differ in some of the processes immediately related to the pathogenesis of hypertension, such as sodium ion transport, but also in processes involved in cellular and vascular remodeling that point toward indirect effects of RDN on the vasculature and other organs and to organ damage being a determinant of response.

Our study adds to other studies that failed to describe clinically meaningful predictors of the response to RDN. The following limitations should be taken into consideration. First, our study is underpowered to show associations with modest BP effects, but we are confident that we did not miss a clinically relevant association that would guide individual treatment decisions. More subtle associations that could provide pathophysiological clues, irrespective of immediate clinical relevance, cannot be excluded due to limited power; however, the gene ontology analyses we conducted do provide such clues. Single genetic variants, confirmed in meta-analyses of large genome-wide association studies, demonstrate BP effects of 0.5 to 1 mm Hg per variant,^23^ and the effect might well be larger in a study measuring the response to a precise and invasive intervention. Second, baseline BP is the key predictor of the response to any antihypertensive treatment^18,28^ and has to be taken into account when absolute or relative responses are analyzed. In our study we decided to define response based on the median response, as assessed by the correlation between ambulatory BP change and baseline ambulatory BP. There are other definitions of BP response that could have been chosen but in general, the 6-month response of BP to RDN comes with the advantage that antihypertensive medication remained unchanged during this period. Also, a sensitivity analysis with more stringent definitions of response resulted in similar results. Third, the wide variability of BP response to RDN challenges any study into possible predictors. We have therefore selected a large cohort of patients who underwent RDN in only 2 clinical centers by experienced interventionists. This process should have reduced operator- and center-related variation to a minimum and should also result in as complete RDN as technically possible.

Genome-wide analyses should not be liberally used to investigate associations between single variants and the phenotype of interest. When we explored our dataset for known variants of genes that could be hypothesized to be involved in SNS activity or response to RDN, we also found no statistically significant association. We conclude from our data that studies investigating genetic determinants of the response to RDN should not be of any priority in the foreseeable future.

We are aware that our study only included patients who underwent radiofrequency RDN with one particular system (Symplicity), whereas other methods, such as ultrasound and alcohol-based RDN, are also used in clinical practice or trials. We cannot exclude that data could be different for other forms of RDN. However, the basic mechanism of action across all methods of RDN is similar, which is also evidenced by the broadly similar BP-lowering effects irrespective of the method used.^1^ We are therefore confident that similar results would be found in genetic studies of other forms of RDN but appreciate that such studies will have to be conducted. This may also apply to other forms of radiofrequency catheters developed more recently. Further limitations of our study include the retrospective nature of the analysis, lack of formal assessment of adherence to antihypertensive drugs, and the inclusion of patients of exclusively white European background. We appreciate that exclusion of patients with accessory renal arteries is not in line with current clinical and trial practice, but it was the standard at the time the procedures were performed in our patients. These limitations may have implications for the generalization of our findings.

In summary, our data do not support the use of genetic makeup to predict BP response. We found, however, different regulation of biologic processes and cellular/molecular functions that could provide insights into the mechanisms of RDN. For now, adhering to guidelines on patient selection, patient work-up, shared decision-making and the highest possible standard in conducting the procedure, including appropriate aftercare appears advisable.

## Perspectives

Competency in Medical Knowledge: Catheter-based RDN is a therapeutic option for patients with treatment-resistant hypertension.

Competency in patient care: it is currently not possible to predict an individual’s BP response to RDN.

Translational outlook 1: this study has not found an association between genetic variants and BP response to RDN.

Translational outlook 2: this study does not support genetic testing to aid decisions about RDN in the management of patients with treatment-resistant hypertension.

## Article Information

### Sources of Funding

This genetic analysis was supported by an unrestricted extramural grant from Medtronic, Inc., USA. C. Delles is supported by the British Heart Foundation (Center of Research Excellence award RE/18/6/34217). F. Mahfoud is supported by Deutsche Gesellschaft für Kardiologie, Deutsche Forschungsgemeinschaft (Sonderforschungsbereich TRR219, Project-ID 322900939), and Deutsche Herzstiftung.

### Disclosures

F. Mahfoud’s institution (Saarland University) has received scientific support from Ablative Solutions, Medtronic, and ReCor Medical. He has received speaker honoraria and consulting fees from Ablative Solutions, Amgen, AstraZeneca, Bayer, Boehringer Ingelheim, Inari, Medtronic, Merck, ReCor Medical, Servier, and Terumo. R. E. Schmieder’s institution (University Hospital Erlangen) has received scientific research support from Ablative Solutions, Medtronic, and ReCor Medical. He has received speaker honoraria and consulting fees from Ablative Solutions, Medtronic, and ReCor Medical. The other authors report no conflicts.

### Supplemental Material

Tables S1–S4

Figures S1–S7

Reference 30

## Supplementary Material


