# Supplementary material for: Response of Blood Pressure to Renal Denervation Is Not Associated With Genetic Variants
Source: Hypertension. 2024 Nov 21;82(1):118–25. doi: 10.1161/HYPERTENSIONAHA.124.23393 (PMC11654447; doi:10.1161/HYPERTENSIONAHA.124.23393)
Supplement: Supplementary file 1 [file hyp-82-118-s002.pdf]

RESPONSE OF BLOOD PRESSURE TO RENAL DENERVATION IS NOT ASSOCIATED WITH GENETIC VARIANTS

Short title: Genetics and renal denervation

Christian Delles[1], Roland E. Schmieder[2], Rónán Daly[3], Dennis Kannenkeril[2], Agnes Bosch[2], Lucas Lauder[4,5], Michael Kunz[4,5], Michael Böhm[4], Graham Hamilton[3], Raphael S. Schmieder[6], Axel Schmid[6], Pawel Herzyk[3], Felix Mahfoud[4,5,7]

[1] School of Cardiovascular and Metabolic Health, University of Glasgow, Glasgow, UK

[2] Department of Nephrology and Hypertension, University Hospital Erlangen, Friedrich-Alexander University Erlangen-Nürnberg, Erlangen, Germany

[3] Glasgow Polyomics, Wolfson Wohl Cancer Research Centre, University of Glasgow, Glasgow, UK

[4] Department of Internal Medicine III, Cardiology, Angiology, Intensive Care Medicine, Saarland University Hospital, Homburg, Germany

[5] Department of Cardiology, University Heart Center Basel, University Hospital Basel, Switzerland

[6] Institute of Radiology, University Hospital Erlangen, Friedrich-Alexander University Erlangen-Nürnberg, Erlangen, Germany

[7] Institute for Medical Engineering and Science, Massachusetts Institute of Technology, Cambridge, Massachusetts, United States of America

Reference

1. McLaren W, Gil L, Hunt SE, Riat HS, Ritchie GR, Thormann A, Flicek P, Cunningham F. The Ensembl Variant Effect Predictor. *Genome Biol*. 2016;17:122. doi: 10.1186/s13059-016-0974-4

Table S1. Blood Pressure Changes in Responders and Non-responders.

	**Responder N=134**	SD	**Non-Responder N=134**	SD	p-value
**Excess** Δ SBP 6months (24 h amb)	-13.3	9.9	11.9	10.4	< 0.005
ΔSBP 6 months (**measured**) (24 h amb)	-23.3	15.9	1.4	11.0	< 0.005
ΔDBP 6 months (**measured**) (24 h amb)	-12.6	10.7	0.4	6.5	< 0.005

The table displays changes in systolic and diastolic 24-h ambulatory blood pressure between baseline and 6-month follow-up. "Excess Δ SBP" indicates the change beyond the mean response predicted from the regression between baseline systolic 24-h ambulatory blood pressure and change in systolic 24-h ambulatory blood pressure as shown in Supplementary Figure 1. All blood pressure data are given in mmHg.

Table S2. Gene Ontology Analysis of Biological Processes

TermID	Name	Value	LogSize	Frequency	Uniqueness	Dispensability
GO:0001578	microtubule bundle formation	-2.557601319	2.130333768	0.749273093	0.866510269	0.16839598
GO:0003356	regulation of cilium beat frequency	-1.395205374	1.255272505	0.095057034	0.996834665	0.03159701
GO:0006631	fatty acid metabolic process	-1.595757421	2.5132176	1.817266831	0.896054559	0.57852323
GO:0006814	sodium ion transport	-1.383989392	2.33243846	1.196600313	0.975434712	0.21830706
GO:0006869	lipid transport	-1.311475129	2.547774705	1.968239767	0.974970761	0.30077163
GO:0007155	cell adhesion	-7.29787228	2.983626287	5.379109819	0.991598902	0.00936298
GO:0007156	homophilic cell adhesion via plasma membrane adhesion molecules	-13.33321815	2.225309282	0.933795571	0.877424654	0
GO:0098742	cell-cell adhesion via plasma-membrane adhesion molecules	-9.512082148	2.423245874	1.476179826	0.872965657	0.7579406
GO:0007411	axon guidance	-1.807508371	2.352182518	1.252516216	0.722875682	0.17852266
GO:0097485	neuron projection guidance	-1.963880967	2.354108439	1.258107806	0.722752135	0.80069499
GO:0007600	sensory perception	-2.83161275	2.996511672	5.541265936	0.883969371	0.69684887
GO:0007608	sensory perception of smell	-1.322728146	2.668385917	2.600089465	0.892663232	0.14056467
GO:0009653	anatomical structure morphogenesis	-2.938394497	3.351409752	12.55312011	0.925304434	0.26820696
GO:0009812	flavonoid metabolic process	-1.646707498	1.113943352	0.067099083	0.98032039	0.07086141
GO:0009888	tissue development	-2.253802682	3.248708736	9.90829792	0.927511008	0.39465115
GO:0030030	cell projection organization	-3.040118462	3.086715664	6.821740103	0.894692413	0.22293536
GO:0030198	extracellular matrix organization	-4.953042285	2.445604203	1.554462089	0.911033889	0.00750789
GO:0031589	cell-substrate adhesion	-2.024693316	2.267171728	1.028852606	0.882556953	0.68680608
GO:0032501	multicellular organismal process	-1.354313588	3.797475288	35.07045404	1	0
GO:0033627	cell adhesion mediated by integrin	-1.786550303	1.568201724	0.201297249	0.897014375	0.50826741

GO:0035082	axoneme assembly	-1.928377211	2.017033339	0.575933796	0.79513772	0.66311786
GO:0070286	axonemal dynein complex assembly	-1.478533131	1.62324929	0.2292552	0.809597406	0.86464515
GO:0035239	tube morphogenesis	-1.653996177	2.833147112	3.802281369	0.867013151	0.47445673
GO:0042632	cholesterol homeostasis	-1.416129424	1.959041392	0.503243122	0.939791288	0.40698589
GO:0055092	sterol homeostasis	-1.36108365	1.963787827	0.508834713	0.939772934	0.87702424
GO:0043062	extracellular structure organization	-5.154760453	2.447158031	1.560053679	0.910999413	0.18338927
GO:0044782	cilium organization	-1.643586862	2.58546073	2.147170655	0.806026183	0.61976467
GO:0120036	plasma membrane bounded cell projection organization	-3.164799825	3.068927612	6.547752181	0.792650874	0.76194985
GO:0045005	DNA-templated DNA replication maintenance of fidelity	-1.82548774	1.763427994	0.318720644	0.942114254	0.06805186
GO:0006261	DNA-templated DNA replication	-1.46357861	2.164352856	0.810780586	0.940562128	0.83731654
GO:0045229	external encapsulating structure organization	-5.257152128	2.44870632	1.56564527	0.910965036	0.18331013
GO:0045494	photoreceptor cell maintenance	-2.683208091	1.643452676	0.240438381	0.910204183	0
GO:0050877	nervous system process	-1.506538796	3.172894698	8.320286289	0.884701516	0.69845132
GO:0050982	detection of mechanical stimulus	-1.656842666	1.748188027	0.307537464	0.99245936	0.09531722
GO:0051056	regulation of small GTPase mediated signal transduction	-2.285095629	2.485721426	1.705435026	0.996428395	0
GO:0051606	detection of stimulus	-1.531562044	2.831869774	3.791098188	0.991780683	0.07764458
GO:0052697	xenobiotic glucuronidation	-3.517071973	0.903089987	0.039141132	0.872936192	0.00529997
GO:0052695	cellular glucuronidation	-2.465287681	1.322219295	0.111831805	0.87425109	0.89656342
GO:0052696	flavonoid glucuronidation	-3.449915026	0.77815125	0.027957951	0.881164603	0.84706013
GO:0070254	mucus secretion	-1.392972199	1.204119983	0.083873854	0.929228402	0.10375709
GO:0072359	circulatory system development	-1.813585354	2.967547976	5.18340416	0.874253573	0.35219795
GO:0098609	cell-cell adhesion	-2.904448711	2.743509765	3.092149407	0.871604849	0.67863669
GO:1904683	regulation of metalloendopeptidase activity	-1.869282485	0.698970004	0.022366361	0.997357711	0.02217746

Table S3. Gene Ontology Analysis of Cellular Components

TermID	Name	Value	LogSize	Frequency	Uniqueness	Dispensability
GO:0001750	photoreceptor outer segment	-2.184273475	1.982271233	0.500869932	0.742068051	0.61554079
GO:0097731	9+0 non-motile cilium	-1.519499569	2.123851641	0.69594559	0.739012056	0.91042366
GO:0097733	photoreceptor cell cilium	-2.075473606	2.08278537	0.632677809	0.736895503	0.95237439
GO:0005581	collagen trimer	-2.541867573	1.944482672	0.458691411	0.955601301	0.14408318
GO:0005593	FACIT collagen trimer	-1.518123523	0.84509804	0.03163389	0.963289827	0.116296
GO:0005775	vacuolar lumen	-1.446463182	2.250420002	0.933199768	0.957281163	0.19342038
GO:0005788	endoplasmic reticulum lumen	-2.673426223	2.496929648	1.650234618	0.962427925	0.03754327
GO:0005856	cytoskeleton	-3.484623284	3.390228362	12.94353351	0.931126902	0.27063211
GO:0005886	plasma membrane	-8.599483654	3.779235632	31.70770285	0.86549684	0.30233725
GO:0005903	brush border	-1.596810054	2.037426498	0.569410028	0.945307609	0.00001369
GO:0031526	brush border membrane	-1.463958676	1.785329835	0.316338904	0.671380679	0.85319062
GO:0032421	stereocilium bundle	-1.445104997	1.851258349	0.369062055	0.946330543	0.86540199
GO:0005911	cell-cell junction	-1.603073827	2.720159303	2.762693099	0.968401114	0.00001674
GO:0005929	cilium	-4.480035599	2.896526217	4.149311963	0.765832162	0.51698242
GO:0005930	axoneme	-5.940748005	2.276461804	0.991195234	0.671164652	0.00001463
GO:0005858	axonemal dynein complex	-3.46048051	1.361727836	0.115990932	0.68542383	0.80235877
GO:0031514	motile cilium	-2.116134263	2.501059262	1.666051563	0.736047889	0.70048972
GO:0032838	plasma membrane bounded cell projection cytoplasm	-2.399467065	2.442479769	1.45515896	0.747449952	0.91412933
GO:0097014	ciliary plasm	-6.203315632	2.278753601	0.996467549	0.718364601	0.9039632
GO:0005938	cell cortex	-2.100825945	2.509202522	1.697685454	0.897049502	0.18573633
GO:0009925	basal plasma membrane	-1.338338934	2.475671188	1.571149892	0.845840331	0.60862576
GO:0015630	microtubule cytoskeleton	-1.442903606	3.1532049	7.497232035	0.908725314	0.49410692
GO:0016020	membrane	-2.392497745	3.990383259	51.56324142	0.999952202	0.00006858
GO:0016324	apical plasma membrane	-2.89720602	2.610660163	2.145832235	0.840884942	0.63292158
GO:0016342	catenin complex	-1.599856756	1.505149978	0.163441767	0.879574061	0.14198099

GO:0016459	myosin complex	-1.802699586	1.770852012	0.305794274	0.898914997	0.34216247
GO:0030018	Z disc	-2.366614189	2.123851641	0.69594559	0.856971374	0.18807035
GO:0031674	I band	-1.5771928	2.167317335	0.769758001	0.855722794	0.89875637
GO:0030286	dynein complex	-1.579164158	1.740362689	0.284705014	0.885158386	0.33982839
GO:0030312	external encapsulating structure	-9.041061948	2.751279104	2.968313386	0.904317315	0.20048537
GO:0031012	extracellular matrix	-8.802099674	2.750508395	2.963041071	0.8333354	0
GO:0005604	basement membrane	-6.78383266	1.968482949	0.485052987	0.855774439	0.77145604
GO:0062023	collagen-containing extracellular matrix	-7.439151607	2.632457292	2.256550851	0.837505217	0.93369127
GO:0031253	cell projection membrane	-4.869158017	2.556302501	1.892761111	0.672243061	0.43072259
GO:0031256	leading edge membrane	-3.794913405	2.264817823	0.964833658	0.866584907	0.17287431
GO:0001726	ruffle	-1.356575685	2.269512944	0.975378289	0.770581646	0.7978267
GO:0032587	ruffle membrane	-1.904936967	2.012837225	0.537776138	0.677040529	0.7496825
GO:0031965	nuclear membrane	-1.343623918	2.498310554	1.655506933	0.949792825	0.24833833
GO:0032982	myosin filament	-1.687007661	1.397940009	0.126535562	0.925393386	0.467013
GO:0036457	keratohyalin granule	-1.316262924	0.698970004	0.02108926	0.990451134	0.02357318
GO:0042734	presynaptic membrane	-1.539604656	2.269512944	0.975378289	0.825961812	0.57487597
GO:0042995	cell projection	-5.957684679	3.385963571	12.81699794	0.999963784	0.00003805
GO:0043194	axon initial segment	-1.553195313	1.380211242	0.121263247	0.831530724	0.32625139
GO:0043202	lysosomal lumen	-1.479166727	1.995635195	0.516686877	0.958730525	0.59218867
GO:0043235	receptor complex	-1.611115026	2.736396502	2.8681394	0.948674933	0.18045683
GO:0043256	laminin complex	-1.583525697	1	0.047450836	0.850457729	0.61094143
GO:0045177	apical part of cell	-3.357501487	2.675778342	2.49380503	0.999971768	0.0000165
GO:0045178	basal part of cell	-1.977625983	2.50242712	1.671323878	0.999973193	0.00001563
GO:0070161	anchoring junction	-1.380616683	2.961421094	4.818895977	0.967384595	0.54638508
GO:0071944	cell periphery	-9.628113635	3.812378511	34.22259714	0.999956216	0.00002591
GO:0097730	non-motile cilium	-2.601915924	2.23299611	0.896293563	0.75113799	0.65393816
GO:0098590	plasma membrane region	-5.727773781	3.124178055	7.012179048	0.884694976	0.37062327
GO:0098636	protein complex involved in cell adhesion	-4.814215103	1.763427994	0.300521959	0.957000787	0

GO:0098637	protein complex involved in cell-matrix adhesion	-2.664501896	1.255272505	0.089629356	0.937291118	0.12574007
GO:0008305	integrin complex	-1.683268868	1.505149978	0.163441767	0.857270319	0.86200336
GO:0098644	complex of collagen trimers	-1.652831678	1.342422681	0.110718617	0.808508218	0.66107751
GO:0098858	actin-based cell projection	-2.051277838	2.372912003	1.238994042	0.799472709	0.4422221
GO:0098862	cluster of actin-based cell projections	-3.618651528	2.238046103	0.906838193	0.999975105	0.00001447
GO:0099080	supramolecular complex	-1.966070452	3.159867847	7.613222966	0.999966798	0.00003261
GO:0099512	supramolecular fiber	-3.047142256	3.027757205	5.615015553	0.897082704	0.69061366
GO:0099081	supramolecular polymer	-3.119139436	3.031004281	5.657194074	0.904333426	0.82727216
GO:0099568	cytoplasmic region	-3.430845464	2.495544338	1.644962303	0.986053086	0.0353489
GO:0120025	plasma membrane bounded cell projection	-5.337863931	3.364175633	12.18959245	0.749281828	0.677567

Table S4. Gene Ontology Analysis of Molecular Function

TermID	Name	Value	LogSize	Frequency	Uniqueness	Dispensability	Representative
GO:0003774	cytoskeletal motor activity	-3.234901786	2.06069784	0.623461854	1	0	null
GO:0004114	3',5'-cyclic-nucleotide phosphodiesterase activity	-1.304485866	1.397940009	0.131255127	0.916857687	0.56171572	null
GO:0004713	protein tyrosine kinase activity	-4.289774188	2.155336037	0.776592836	0.89038478	0	null
GO:0004714	transmembrane receptor protein tyrosine kinase activity	-2.203139406	1.799340549	0.339075745	0.884673313	0.63410145	null
GO:0005001	transmembrane receptor protein tyrosine phosphatase activity	-1.390926182	1.255272505	0.092972382	0.876290933	0.30254953	null
GO:0005044	scavenger receptor activity	-3.35727492	1.447158031	0.147662018	0.887855762	0.40206486	null
GO:0005085	guanyl-nucleotide exchange factor activity	-5.302322515	2.369215857	1.274268526	0.919007178	0	null
GO:0030695	GTPase regulator activity	-2.023605947	2.697229343	2.718074925	0.915606512	0.88032971	5085
GO:0005201	extracellular matrix structural constituent	-6.824521927	2.222716471	0.907847963	0.968940344	0	null
GO:0005319	lipid transporter activity	-3.615671904	2.247973266	0.962537599	0.77220653	0.24713561	null
GO:0005509	calcium ion binding	-7.941475245	2.854913022	3.910308996	0.958019326	0	null
GO:0005515	protein binding	-2.615361724	4.15730563	78.5561936	0.996800331	0.00553101	null
GO:0005543	phospholipid binding	-2.07588534	2.693726949	2.69619907	0.998231138	0.0026566	null
GO:0008092	cytoskeletal protein binding	-1.394057851	2.999565488	5.458025704	0.995210906	0.01616627	null
GO:0008289	lipid binding	-2.018271727	2.937517892	4.730653541	0.998070422	0.00289395	null
GO:0008569	minus-end-directed microtubule motor activity	-4.872890428	1.255272505	0.092972382	0.947906038	0	null
GO:0015081	sodium ion transmembrane transporter activity	-2.177335873	2.220108088	0.902378999	0.775730599	0.65280214	null
GO:0019198	transmembrane receptor protein phosphatase activity	-1.390926182	1.255272505	0.092972382	0.876290933	0.65544163	null

GO:0019199	transmembrane receptor protein kinase activity	-2.755483642	1.913813852	0.442986054	0.882790726	0.65059441	null
GO:0019838	growth factor binding	-2.309258675	2.133538908	0.73831009	0.996126902	0.00929284	null
GO:0022804	active transmembrane transporter activity	-1.391987107	2.664641976	2.521192234	0.773105442	0.60186305	null
GO:0030020	extracellular matrix structural constituent conferring tensile strength	-4.363909069	1.662757832	0.246103363	0.968940344	0.56348372	null
GO:0032559	adenyl ribonucleotide binding	-2.510220676	3.184123354	8.351107465	0.872069789	0.42035653	null
GO:0005524	ATP binding	-1.92740793	3.172018809	8.121410993	0.871863375	0.88389617	32559
GO:0030554	adenyl nucleotide binding	-2.289314191	3.210586025	8.876127974	0.897914007	0.80926684	32559
GO:0034040	ATPase-coupled lipid transmembrane transporter activity	-1.594088128	1	0.049220673	0.782500583	0.62817762	null
GO:0038024	cargo receptor activity	-5.474017228	1.86923172	0.399234345	0.879837209	0	null
GO:0042910	xenobiotic transmembrane transporter activity	-1.346592154	1.51851394	0.175006836	0.815313018	0.45578888	null
GO:0043394	proteoglycan binding	-2.75548454	1.568201724	0.196882691	0.950793307	0.0019226	null
GO:0048407	platelet-derived growth factor binding	-1.527144937	1.079181246	0.0601586	0.959317378	0.69138971	null
GO:0050431	transforming growth factor beta binding	-2.502838292	1.380211242	0.125786164	0.959126744	0.00801879	null
GO:0050839	cell adhesion molecule binding	-1.368543615	2.745074792	3.035274815	0.995512397	0.01231781	null
GO:0060589	nucleoside-triphosphatase regulator activity	-2.023605947	2.697229343	2.718074925	0.93012354	0.60831995	null
GO:0099604	ligand-gated calcium channel activity	-2.209765412	1.51851394	0.175006836	0.789223332	0.45578888	null
GO:0099094	ligand-gated monoatomic cation channel activity	-1.486434092	2.139879086	0.749248018	0.770758026	0.80794097	99604

GO:0140326	ATPase-coupled intramembrane lipid transporter activity	-2.70371473	1.447158031	0.147662018	0.770930121	0.69000916	null
GO:0140657	ATP-dependent activity	-2.337930061	2.767897616	3.199343724	1	0	null

Tables S2 to S4 display data from analysis with REVIGO (http://revigo.irb.ht). Raw data are displayed. Value, -log10 (p-value); LogSize, log10 (number of annotations for GO term ID in H. sapiens in the EBI GOA database); Frequency, proportion of the GO term in the underlying (H. sapiens) protein annotation database; Uniqueness, measures whether the term is an outlier when compared semantically to the whole list; Dispensability, semantic similarity threshold at which the term was removed from the list and assigned to a cluster.

Figure S1. Definition of Responders and Non-responders.

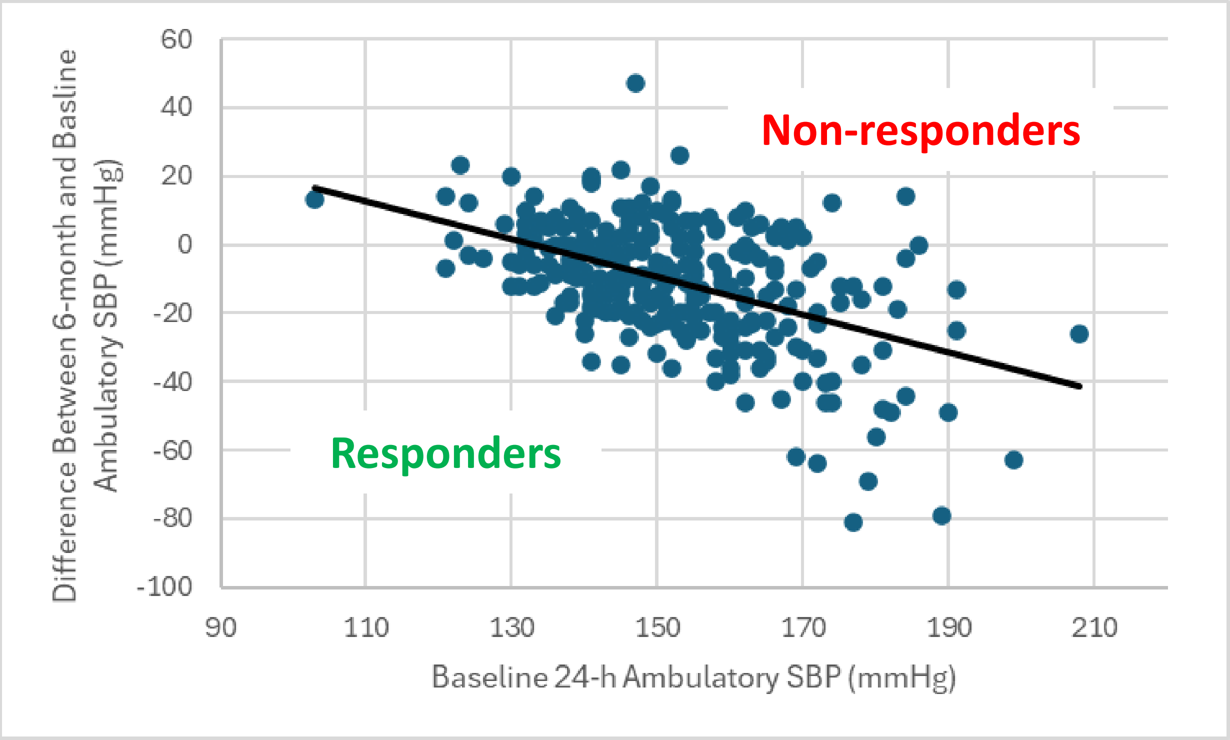

Change in 24-h ambulatory systolic blood pressure (SBP) between baseline visit and 6-month follow-up (y-axis) is plotted against baseline 24-h ambulatory SBP (x-axis). Linear regression was applied and the regression line (black) is shown. Responders are those whose change in 24-h ambulatory SBP was greater than the mean response indicated by the regression line. Non-responders are those whose change in 24-h ambulatory SBP was smaller than the mean response indicated by the regression line.

Figure S2. Sequencing data analysis pipeline.

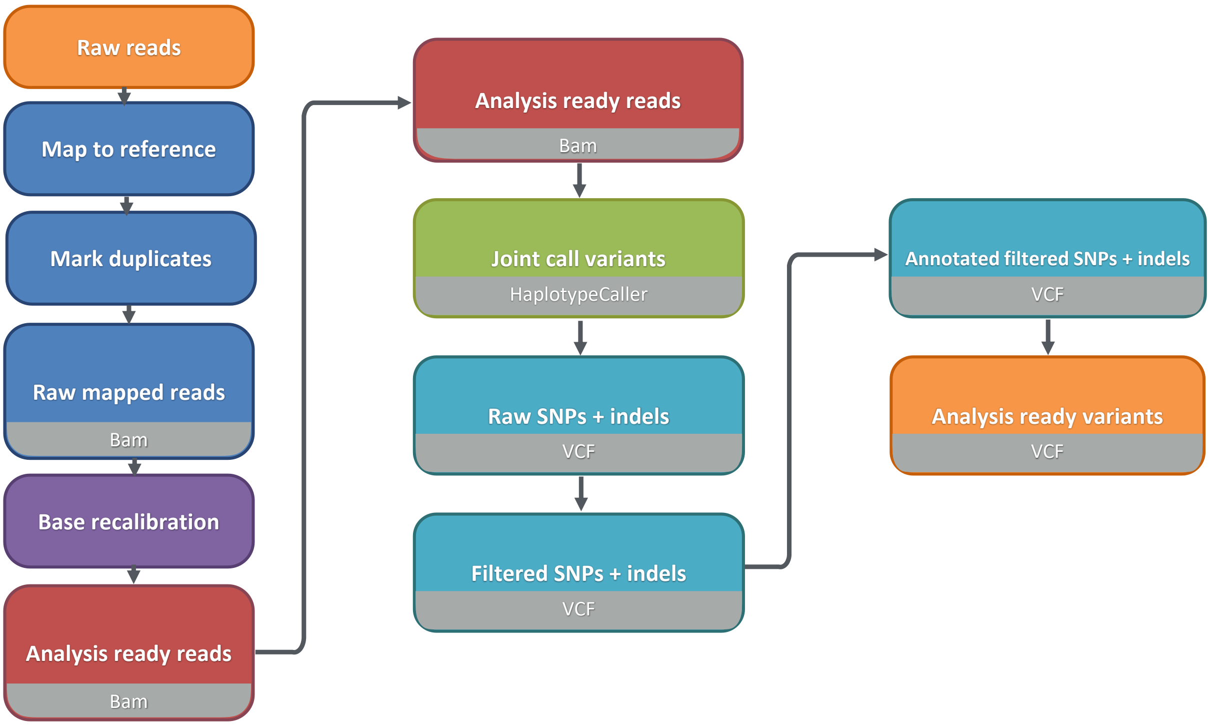

Abbreviations: VCF, Variant Call Format. Bam, compressed binary version of SAM as described in https://samtools.github.io/hts-specs/SAMv1.pdf. Details on HaplotypeCaller can be found on https://gatk.broadinstitute.org/hc/en-us/articles/360037225632-HaplotypeCaller.

Figure S3. Exome Genome Sequencing Coverage.

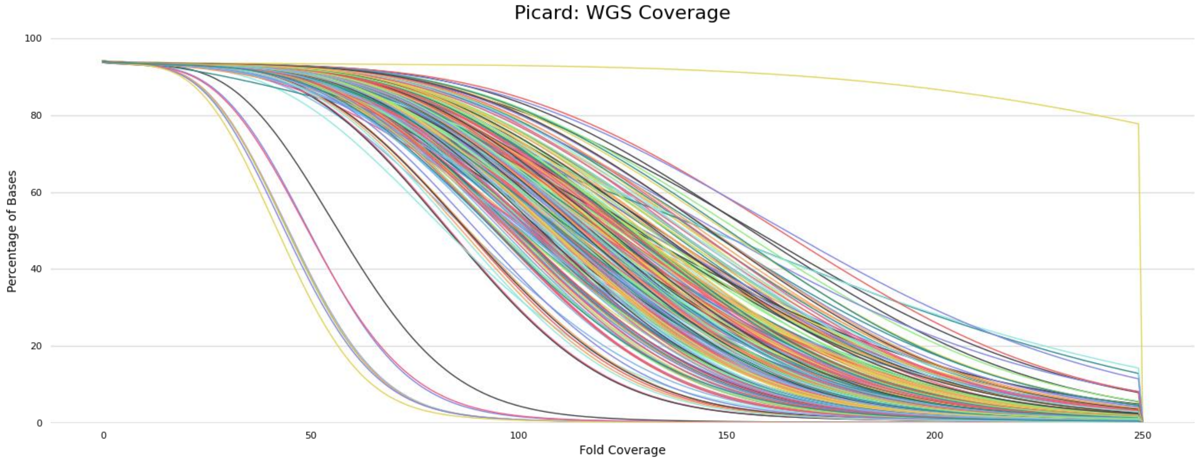

Analysis was conducted using the CollectRawWgsMetrics (Picard) tool as described on https://gatk.broadinstitute.org/hc/en-us/articles/360040508411-CollectRawWgsMetrics-Picard-. Eight samples have lower coverage (s96, s97, s99, s100, s101, s102, s103 & s105). However, they have a coverage of 30 reads per base for 80% of the exome.

Figure S4. Post-hoc Power Analysis.

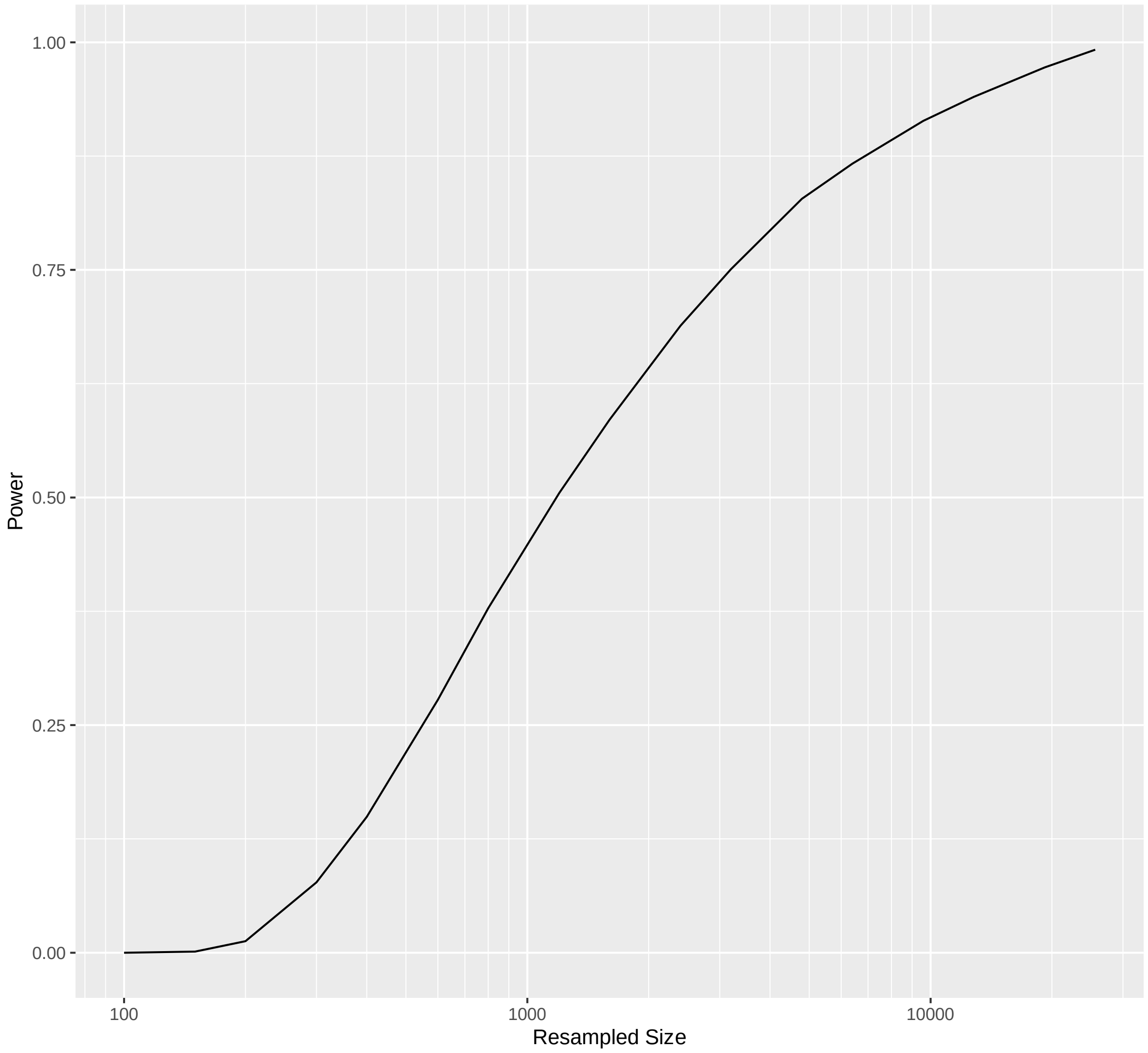

A post-hoc power analysis was conducted by treating the results as a pilot study and resampling the data with replacement, with resample sizes between 100 and 25600. The analysis indicates that given the effects from the measured data, a total sample size of 4219 would be needed for a power of 0.8.

Figure S5. Summary of Variant Annotation.

A

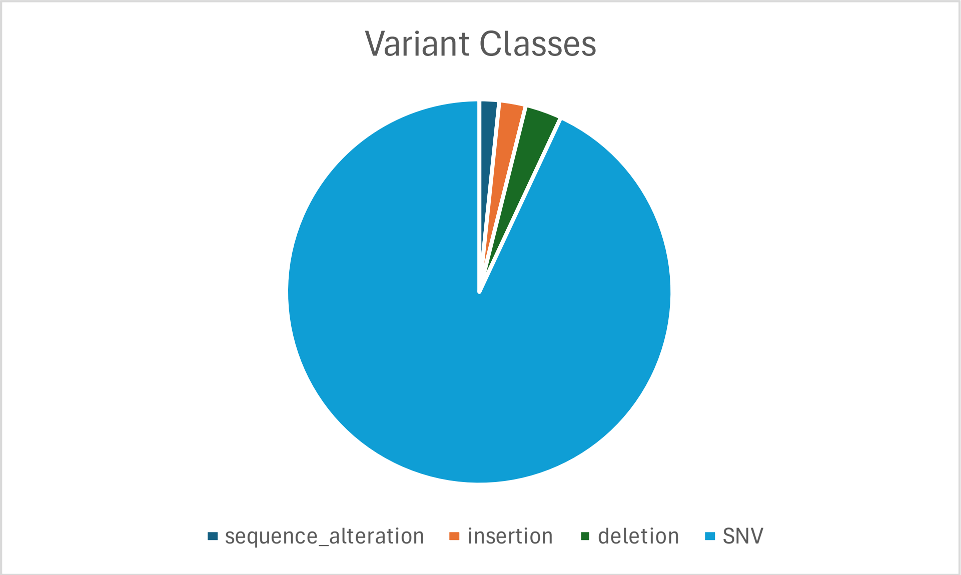

B

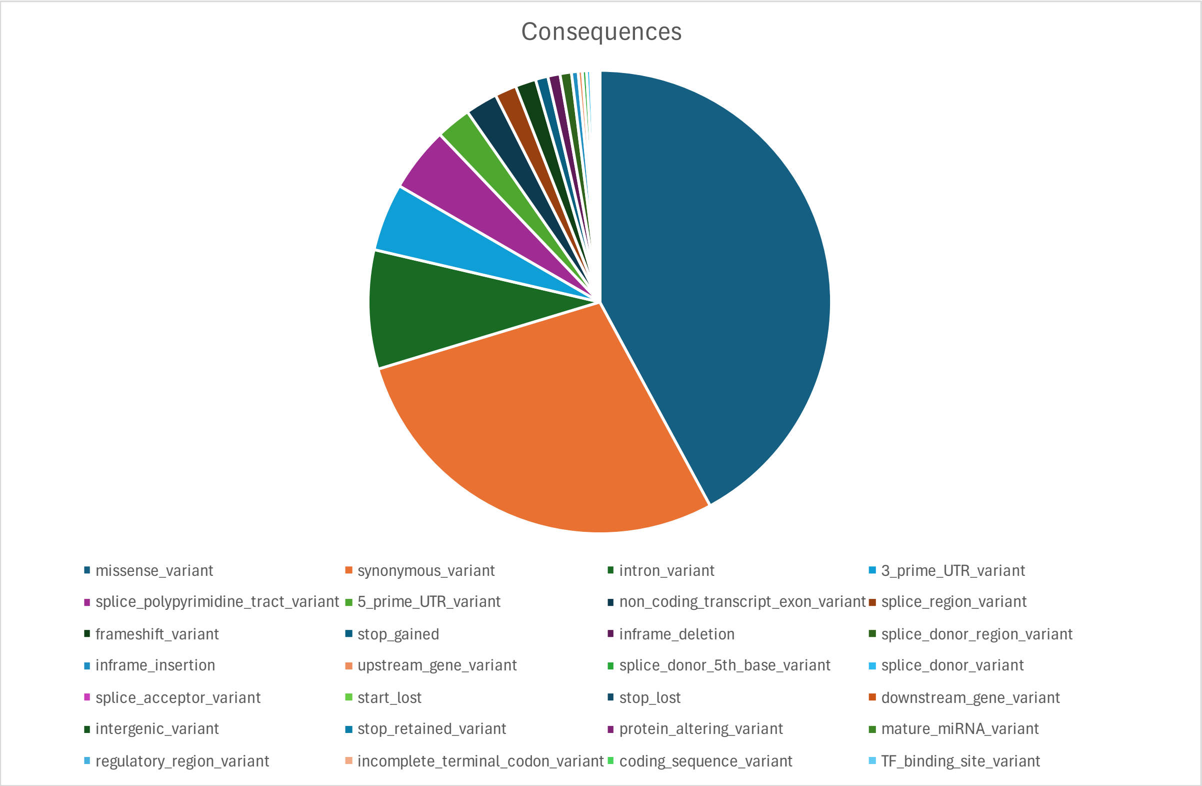

Analysis was conducted using Variant Effect Predictor [1]. A total of 249,669 variants were processed. There were 30,544 overlapped genes, 175,568 overlapped transcripts and 32,153 overlapped regulatory features. A, Pie chart summarising the variant classes identified in the analysis. B, Pie chart summarising the predicted consequences for the variants identified in the analysis.

Figure S6. Sensitivity Analysis. PCA Scatter plot Matrix.

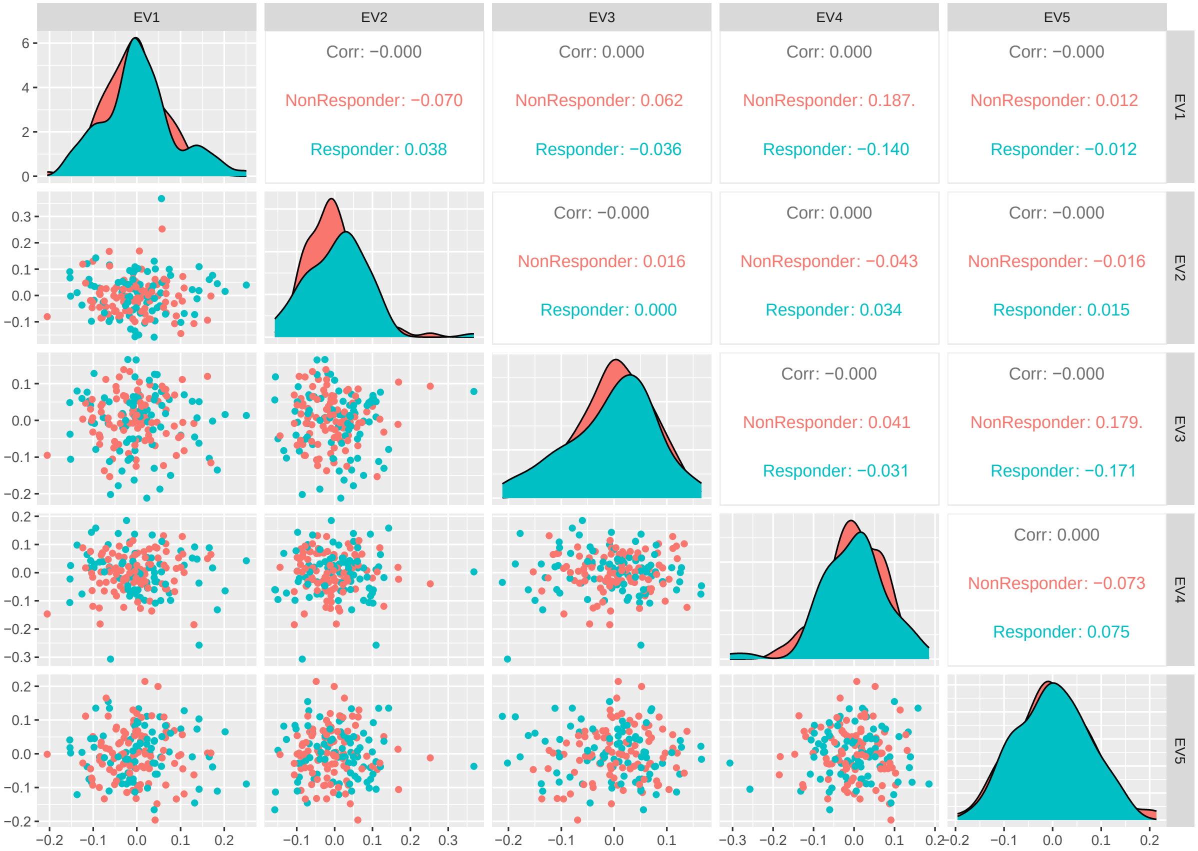

A sensitivity analysis was conducted with a more stringent definition of response *vs* non-response to RDN by comparing the top with the bottom tertile of response. Analysis as per Figure 2 in the main paper. The plot shows no separation between the responders and non-responders over these components. Corr, correlation; EV, eigenvector. Axes are unit-less.

Figure S7. Sensitivity Analysis. Distribution of p-values.

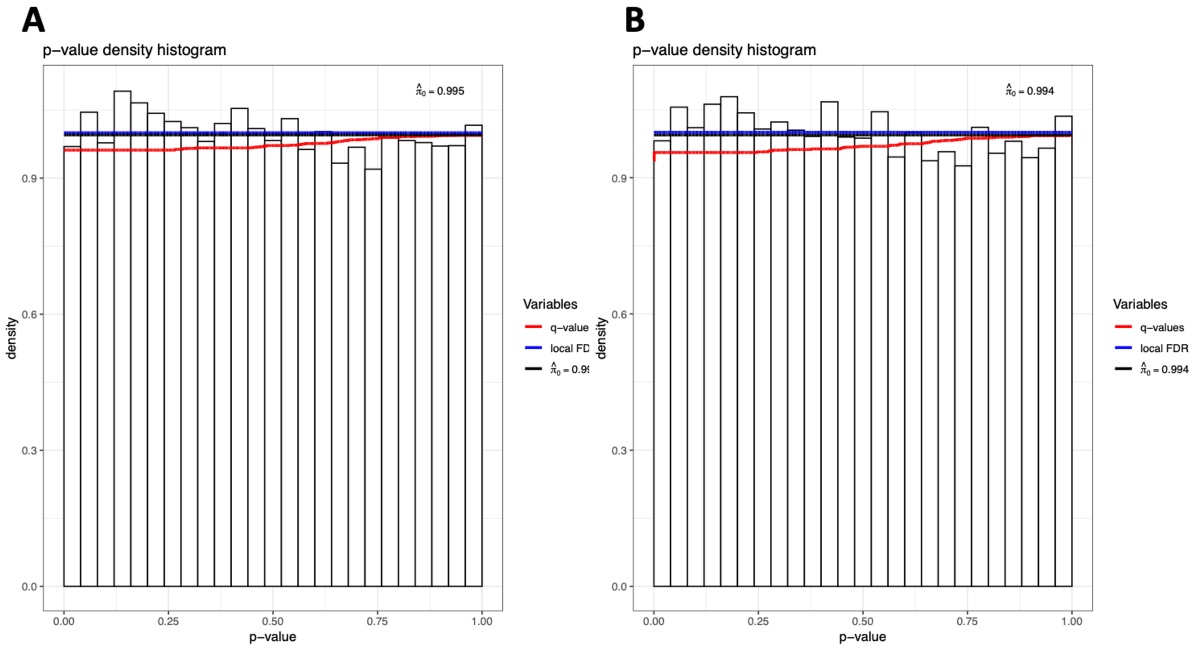

A sensitivity analysis was conducted with a more stringent definition of response *vs* non-response to RDN by comparing the top with the bottom tertile of response. Analysis as per Figure 3 in the main paper. A, Distribution of p-values in unadjusted data. B, Distribution of p-values in data adjusted for sex. Overall no evidence was found to reject the null hypothesis of no genome-wide association with blood pressure response to renal denervation.
